# P-2011. Blood Transcriptomic Stratification for Acute Encephalitis/encephalopathy and Croup in Children with Omicron Infection

**DOI:** 10.1093/ofid/ofae631.2168

**Published:** 2025-01-29

**Authors:** Chong-Wei Huang, Ian Yi-Feng Chang, Wei-Chao Liao, Jainn-Jim Lin, En-Wei Hsing, Chih-Ho Chen, Chen-Yen Kuo, Chih-Jung Chen, Yhu-Chering Huang, Cheng Chiu, Yu-Chia Hsieh

**Affiliations:** Keelung Chang Gung Memorial Hospital, department of Pediatric / Attending, Taoyuan, Taoyuan, Taiwan (Republic of China); Molecular Medicine Research Center, Chang Gung University, Taoyuan, Taoyuan, Taiwan; Molecular Medicine Research Center, Chang Gung University, Taoyuan, Taoyuan, Taiwan; Linkou Chang Gung Memorial hospital, Taoyuan, Taoyuan, Taiwan; Chang Gung University, Taoyuan, Taoyuan, Taiwan; Division of Pediatric Infectious Diseases, Department of Pediatrics, Kaohsiung Chang Gung Memorial Hospital, Kaohsiung, Kaohsiung, Kaohsiung, Taiwan; Division of Pediatric Infectious Diseases, Department of Pediatrics, Chang Gung Memorial Hospital, Taoyuan, Taoyuan, Taiwan; Division of Pediatric Infectious Diseases, Department of Pediatrics, Chang Gung Memorial Hospital, Taoyuan, Taoyuan, Taiwan; Division of Pediatric Infectious Diseases, Department of Pediatrics, Chang Gung Memorial Hospital, Taoyuan, Taoyuan, Taiwan; Chang Gung Memorial Hospital, Taoyuan, Taipei, Taiwan; Linkou Chang Gung Memorial Hospital, Taipei, Taipei, Taiwan

## Abstract

**Background:**

Omicron poses significant concerns, leading to two severe complications, encephalitis and croup in children. Neutrophil predominance and lymphopenia were observed in severe cases. We propose a distinctive blood RNA signature to characterize COVID-19's immune landscape, potentially aiding in differentiating severe from mild cases in pediatric patients, with implications for clinical management.

Volcano plot of differentially expressed genes between encephalitis and mild disease

**Figure:**
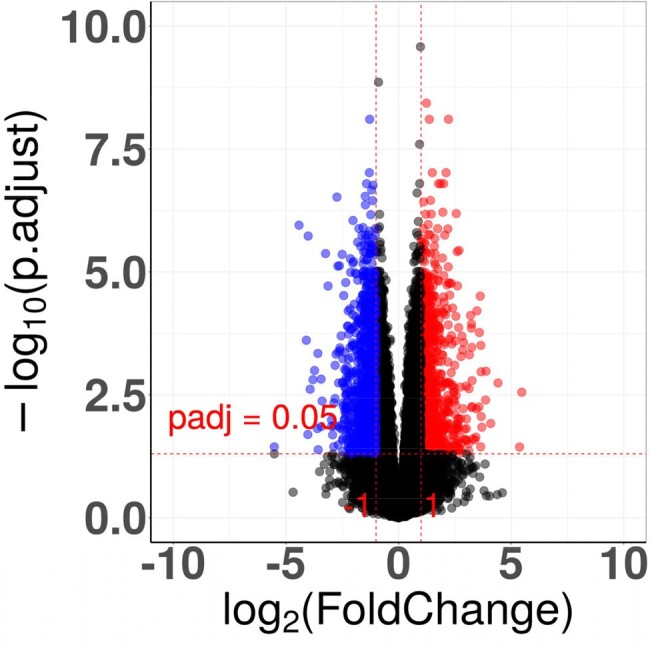

Fold change was the ratio of the average expression of genes in the encephalitis/encephalopathy group to those in the mild disease group. DEGs were determined using cutoff values: |log2 (fold change) | > 1 and adjusted p value < 0.05. Genes with log2(fold change) > 1 and adjusted p value < 0.05 were deemed upregulated, while those with log2(fold change) < -1 and adjusted p value < 0.05 were considered downregulated.

**Methods:**

The case-control cohort spanned two medical centers, Chang Gung Memorial Hospital, Linkou/Kaohsiung branches. Enrolled patients, with confirmed acute SARS-CoV-2 infection, were categorized into mild febrile disease, croup, or encephalitis/encephalopathy. A total of 61 children participated, with 29 mild febrile illness, 14 croup, and 18 encephalitis/encephalopathy cases. Samples collected a median of 2 (1-2) days post-symptom onset underwent RNA sequencing. Differences in gene expression and functional pathways were analyzed. The prediction model for COVID-19-associated encephalitis was developed using random forest analysis.

Encephalitis/encephalopathy vs. mild disease DEGs heatmap

**Figure:**
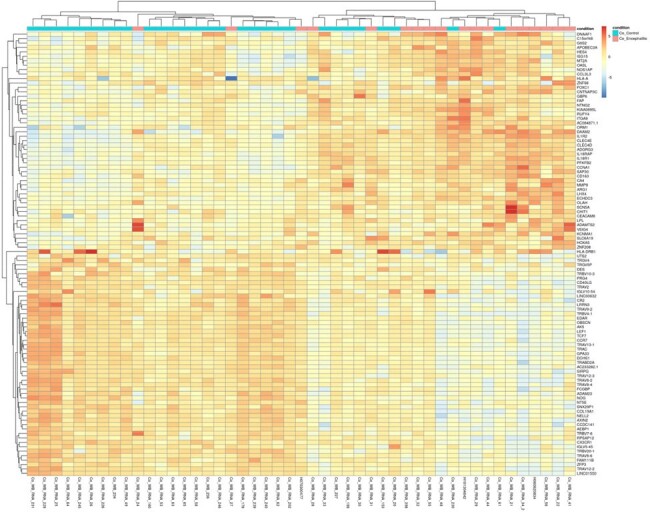

The warmer color indicates a higher expression level of DEGs, whereas cooler colors signify a lower expression level of DEGs.

**Results:**

A total of 1780 and 136 DEGs were identified in mild disease vs. encephalitis/encephalopathy and mild disease vs. croup, respectively. The heatmap from top DEGs indicates clustering into granulocyte activation, response to IFN-γ, and myeloid cell differentiation. GOBP and KEGG enrichment analyses revealed up-regulation of neutrophil function, TNF-alpha signaling pathway, NF-kappa B complex, and down-regulation of T cell activation in COVID-19 encephalitis group. CIBERSORT analysis showed distinct immunological profiles in the encephalitis group: elevated neutrophils, activated dendritic cells, follicular helper T cells; diminished CD4, CD8 T cells, monocytes, naïve B cells. Overexpression of systemic inflammation in severe COVID-19 may result from impaired bridge between innate immunity and adaptive immunity. The top 10-DEG prediction model for COVID-19 associated encephalitis yielded an AUC of 0.938.

Encephalitis/encephalopathy vs. mild disease upregulated DEG GO_BP dot plot.

**Figure:**
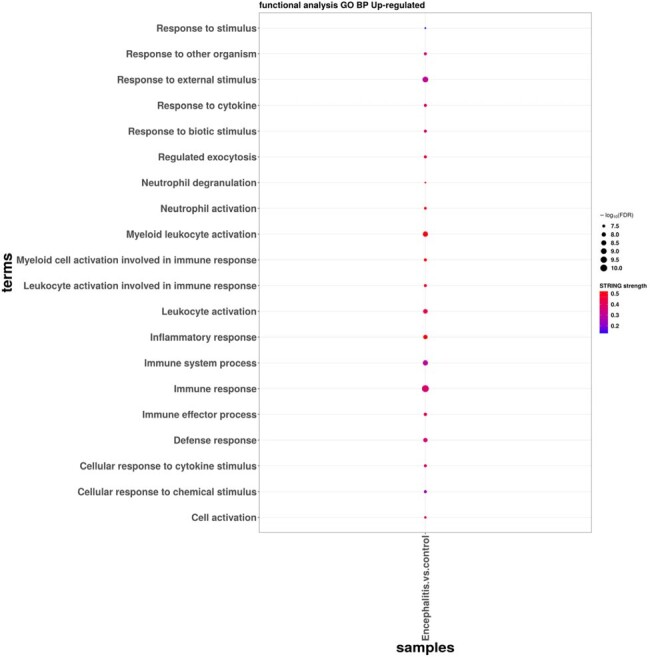

GO is an abbriviation for Gene Ontology, with Biological Process (BP) constituting a pivotal component of GO. Generation refers to the ratio of genes enriched in this biological process to all upregulated DEGs. The shading intensity of the circle indicates the quantity of enriched genes.

**Conclusion:**

In this study, we delineated the immune landscape and functional pathways linked to COVID-19 encephalitis in the pediatric population. Our findings hold potential for enhancing the precision of diagnosing encephalitis/encephalopathy in pediatric COVID-19 patients.

Cibersortx analysis between the encephalitis/encephalopathy group and mild disease group

**Figure:**
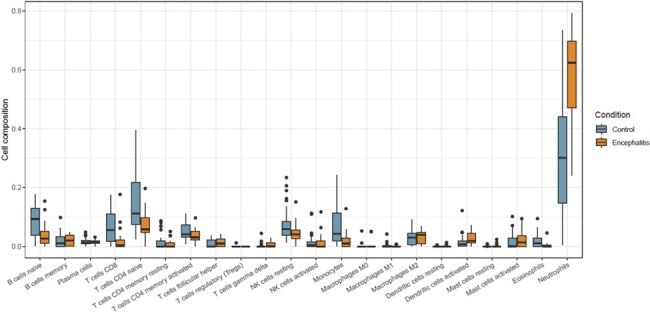

Average percentage of immune cells of the innate immune response and adaptive immune response in the encephalitis/encephalopathy group and mild disease group.

**Disclosures:**

All Authors: No reported disclosures

